# Bromophenolics from the Red Alga *Polysiphonia decipiens*

**DOI:** 10.3390/md17090497

**Published:** 2019-08-26

**Authors:** James Lever, Grace Curtis, Robert Brkljača, Sylvia Urban

**Affiliations:** 1 School of Science (Applied Chemistry and Environmental Science), RMIT University, GPO Box 2476V Melbourne, VIC 3001, Australia; 2 Medicinal Chemistry, Monash Institute of Pharmaceutical Sciences, Monash University (Parkville Campus), 381 Royal Parade, Parkville, VIC 3052, Australia

**Keywords:** *Polysiphonia decipiens*, bromophenolics, marine natural products, structural elucidation

## Abstract

The isolation and the structure determination of a new bromophenolic compound, polysiphonol (**10**), as well as five previously reported compounds, (**4**–**8**), from the red alga *Polysiphonia decipiens* is reported. In addition, the absolute configuration of the natural product rhodomelol (**8**) could be unequivocally confirmed for the first time, and on biosynthetic grounds, the absolute configuration of polysiphonol (**10**) was tentatively suggested. Compounds **4**–**8** were evaluated for their antibacterial activity against both Gram-positive and Gram-negative bacteria, but none of the compounds showed any appreciable activity.

## 1. Introduction

The red alga *Polysiphonia decipiens* is a filamentous red alga belonging to the family Rhodomelaceae and is found predominately around southern coasts of Australia and the islands of New Zealand [1]. The genus *Polysiphonia* contains over 700 species of algae, of which only *P. lanosa* [2,3], *P. ferulacea* [4], *P. sphaerocarpa* [5], *P. urceolata* [6,7,8,9,10], and *P. morrowii* [11] have been phytochemically studied. Studies of the secondary metabolites occurring in these species have revealed the presence of a number of bromophenolic type compounds, including brominated anisoles and cresols, brominated phenanthrenes, bromobenzaldehydes, and benzophenanthrofurans. Monoaromatic benzaldehydes such as 3-bromo-4,5-dihydroxybenzaldehyde (**1**) and 3,5-dibromo-4-hydroxybenzaldehyde (**2**) [9] as well as dibromophenols such as 5,6-dibromoprotocatechualdehyde (**3**) and α-*O*-methyllanosol (**4**) [12] are characteristic classes of compounds of the family Rhodomelaceae. Such compounds have displayed antifeedant [13], antioxidant [7,8,9], and anti-inflammatory [9,11] activities.

**Figure 1:**
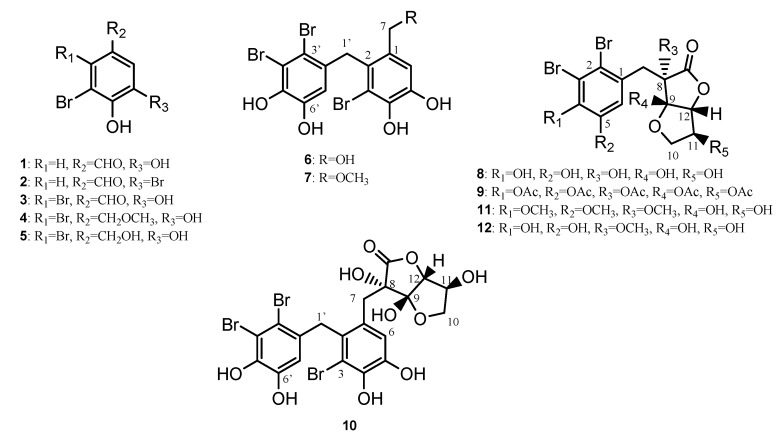
Secondary metabolites from **1**–**10**.

This study marks the first chemical investigation of the secondary metabolites occurring in the red alga *P. decipiens*. Reported is the characterisation of an unprecedented structural derivative of rhodomelol (**8**), (3*R*,3a*R*,6*R*)-3-(3-bromo-2-(2,3-dibromo-4,5-dihydroxybenzyl)-4,5-dihydroxybenzyl) -3,3a,6-trihydroxytetrahydrofuro [3,2-*b*] furan-2(3*H*)-one, which has been given the name polysiphonol (**10**). Also reported is the isolation of five previously described bromophenolics including α-*O*-methyllanosol (**4**), lanosol (**5**), 5-(2-bromo-3,4-dihydroxy-6-(hydroxymethyl)benzyl)-3,4-dibromobenzene-1,2-diol (**6**), 5-(2-bromo-3,4-dihydroxy-6-(methoxymethyl)benzyl)-3,4-dibromobenzene-1,2-diol (**7**), and rhodomelol (**8**) (Figure 1). The first unequivocal absolute stereochemical assignment of the natural product rhodomelol (**8**) is also reported.

## 2. Results and Discussion

### 2.1. Structural Elucidation

The alga was pulverised and extracted using 3:1 MeOH:CH_2_Cl_2_ and then sequentially solvent partitioned (triturated) into CH_2_Cl_2_ and MeOH soluble extracts. Both extracts were analysed using analytical HPLC and ^1^H NMR spectroscopy. The methanol extract was subjected to further purification via C_18_ Vacuum Liquid Chromatography (VLC) (see Section 3.1), which yielded a total of 11 fractions. All fractions were analysed by analytical HPLC equipped with photodiode array (PDA) detection, as well as by ^1^H NMR spectroscopy. Compounds displaying UV maxima at approximately 290 nm were present in a variety of the resulting column fractions. Utilising the taxonomy of the alga and the UV maxima, a search was performed using the MarinLit database [14] in an attempt to expedite the structure elucidation of these compounds. Exact structure confirmation was not achievable at this stage, but it was apparent that the compounds were likely to be bromophenolic type compounds [15,16]. This was based on the ubiquitous nature of these compounds within the genus *Polysiphonia*, supported by the UV maxima (λ_max_: 290 ± 5 nm) of similar bromophenolic type compounds. The 20% methanol/H_2_O (184 mg) C_18_ VLC column fraction was further purified using semi-preparative HPLC (see Section 3.1), which resulted in the isolation of compounds **4**–**8** and **10**. The previously described bromophenolics **4**–**8** were characterised using 1D and 2D NMR spectroscopy, and the data were found to be identical to those previously reported for these compounds [3,16,17,18,19,20]. The relative orientation and the location of the substituents present in compounds **6**–**7**, including the location of the hydroxyl and the methoxy substituents at C1, were confirmed by single irradiation NOE enhancements, which also supported the literature assignments for these compounds [13].

Rhodomelol (**8**) was first isolated from the red alga *P. lanosa* in 1985, but only limited characterisation was carried out on the natural product [3]. Instead, the compound was acetylated, and a proton NMR was reported, but no stereochemistry was reported for this derivative. Methylrhodomelol (**12**) was also isolated from the same *P. lanosa* specimen and was subsequently methylated to yield the derivative (**11**), for which an X-ray crystallographic examination was undertaken. This secured the absolute configuration of (**11**) and therfore methylrhodomelol (**12**). No discussion of the stereochemistry of rhodomelol (**8**) was made, and from this time onwards, only the relative stereostructure has been reported for rhodomelol (**8**). In 1988, a stereoselective synthesis was developed to produce a compound of the same skeletal structure to that of the natural product rhodomelol (**8**) by mixing lanosol (**5**) in the presence L-ascorbic acid [20]. This process has been shown to be reliably stereoselective at 8-OH, 9-OH, 11-OH, and 12-H positions [20,21]. While this study successfully synthesised a product with the same structural skeleton as rhodomelol (**8**) and with known absolute stereochemistry, only ^13^C NMR data and a specific optical rotation measurement were reported. Rhodomelol (**8**) was once again isolated in 2009 from the New Zealand red alga *Osmundaria colensoi* [19]. In this study, rhodomelol (**8**) was reported with complete proton and carbon assignments, but no experimental data were provided for the determination of the relative or the absolute configuration. To date, there has been no reported specific optical rotation of the actual natural product rhodomelol (**8**), meaning that the absolute stereochemistry of this natural product has remained unassigned.

**Figure 2:**
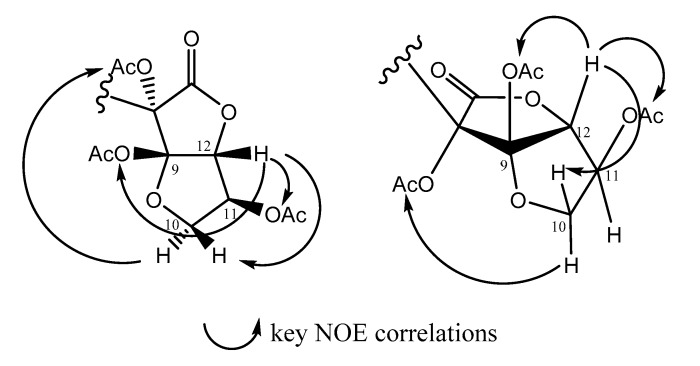
Key NOE correlations for acetylated rhodomelol (**9**).

Reported here is the determination of the relative stereochemistry for rhodomelol (**8**) established via acetylation of **8** followed by analysis of key NOE NMR enhancements. In order to obtain as much information about the orientation of the hydroxyl groups and the relative configuration of the stereocentres present in the lactone ring system of rhodomelol, acetylation was performed (see Section 3.4). Rhodomelol (**8**) (1.8 mg) was combined with a 1:1 mixture of acetic anhydride and pyridine and left to stir for 24 h. The resulting reaction mixture was analysed by both 1D and 2D NMR spectroscopy and confirmed to be the acetylated rhodomelol derivative, compound (**9**). Then, 1D NOE NMR experiments were conducted on compound (**9**) in order to determine the relative configuration at 8-OAc, 9-OAc, 11-OAc, and H12 of the bicyclic lactone moiety. Through-space correlations were observed between H-12 (*δ* 5.54 ppm), 11-OAc (*δ* 2.13 ppm), 9-OAc (*δ* 2.04 ppm), and H-10b (*δ* 4.22 ppm), indicating that these substituents of the bicyclic lactone moiety must be within the same plane. This was further supported by through-space correlations observed between H-10a (*δ* 4.42 ppm) and 8-OAc (*δ* 2.15 ppm), which were on the opposite plane (Figure 2). This suggested the configuration of acetylated rhodomelol (**9**) to be 8*S*, 9*R*, 11*S*, and 12*R*. The specific rotation of the natural product rhodomelol (**8**) was also recorded for the first time (${\left[\alpha \right]}_{D}^{25}$: +29.1 c 0.5, 99% MeOH), compared to synthetically prepared rhodomelol (${\left[\alpha \right]}_{D}^{25}$: +17.4 c 0.5, 95% EtOH), and found to be of the same sign and magnitude. Since the absolute configuration of the synthetically prepared rhodomelol was known [20], this comparison permitted the first unequivocal confirmation of the absolute configuration of the natural product rhodomelol (**8**) to be established as 8*S*, 9*S*, 11*S*, and 12*R*.

The previously unreported bromophenol polysiphonol (**10**) was isolated as a brown oil from the methanol extract. The low resolution ElectroSpray Ionisation (ESI) MS spectrum (negative mode) of polysiphonol (**10**) displayed the [M − H]^−^ ion as a tribrominated peak cluster at *m*/z 653, 655, 657, and 659 (1:3:3:1). This type of isotopic peak cluster is indicative of a compound that contains three bromine atoms. Compound (**10**) was further analysed via 1D (^1^H) and 2D [HSQC with adiabatic pulses (HSQCAD) and gradient HMBCAD (gHMBCAD)] NMR spectroscopic techniques (Table 1).

Initial inspection of the spectroscopic data suggested that polysiphonol (**10**) was closely related to rhodomelol (**8**). This was evident due to the presence of the H-10a/H-10b protons observed at *δ* 4.19 and 4.03 ppm, respectively, which were closely consistent to that of H-10a/10b of rhodomelol (**8**). A broad singlet at *δ* 4.36 ppm that integrated for 2H, accounting for H-11 and H-12, was also observed and was comparable to the broad singlet that rhodomelol displayed at *δ* 4.58 ppm.

The presence of these proton signals supported the fact that polysiphonol (**10**) contained the same bicyclic lactone moiety as that of rhodomelol (**8**). Where polysiphonol differed from rhodomelol was the presence of an additional aromatic ring. Support for the presence of the two aromatic units in the structure of polysiphonol (**10**) was substantiated by comparison of the chemical shifts for the two aromatic singlets in **10** with those in compound **6**. In compound **10**, these occurred at *δ* 5.97 ppm (H-7′) and *δ* 6.92 ppm (H-6), and in the case of compound **6**, these occurred at *δ* 6.06 ppm (H-7′) and *δ* 6.94 ppm (H-6), respectively.

Key gHMBCAD correlations were used to substantiate connectivity of the methylene bridges in the natural product polysiphonol (**10**). Correlations were observed between H-1a’/1b’ and C-2, C-2′, and C-7′, locking in place the methylene between the aromatic rings A and B. This same principle was used to support the connection of ring B to the bicyclic lactone moiety with key correlations being observed between H-7a/7b and C-1 and C-2 from ring B, together with correlations between H-7a/7b and C-8 and C-9 and C-13, providing the basis for the second methylene connection as well as confirming the location of the carbonyl group in the lactone ring.

Due to the instability of polysiphonol (**10**), no further characterisation could be performed. Polysiphonol (**10**) was kept refrigerated in deuterated methanol for four weeks. After this time, it was established by ^1^H NMR that there was significant degradation of this compound. Based on the co-occurrence of **8** and **10** and their structural similarity, on biosynthetic grounds, the absolute configuration of polysiphonol (**10**) is tentatively suggested to be 8S, 9S, 11S, and 12R on the basis of the absolute configuration established for rhodomelol (**8**).

### 2.2. Biological Activity

Previous antibacterial activity was reported for compound **7**, indicating moderate growth inhibition towards an array of Gram-negative (G−) and Gram-positive (G+) bacteria [22]. This included *Staphylococcus aureus* (G+), *Bacillus subtilis* (G+), *Micrococcus luteus* (G+), *Proteus vulgaris* (G−), and *Salmonella typhimurium* (G−), which showed minimum inhibitory concentration (MIC) values of 25 μg/mL in all assays [22]. Compounds **6** and **7** also displayed moderate antibacterial activity when assayed against the Gram-positive *Staphylococcus epidermidis* [23]. Compounds **4**, **5**, and **7** were found to be feeding deterrents to the abalone *Haliotis discus hannai*, while only **7** showed antifeedant activity against the sea urchin *Strongylocentrotus intermedius* [13]. Enzyme inhibition activity was reported for compounds **4** and **5** against α-glucosidase [24], and compound **7** against glucose 6-phosphate dehydrogenase [25].

In the present study, compounds **4**–**8** were all assayed against a range of Gram-positive/negative bacteria [methicillin-resistant *Staphylococcus aureus* (MRSA) (G+), *Escherichia coli* (G−), *Klebsiella pneumoniae* (G−), *Acinetobacter baumannii* (G−), *Pseudomonas aeruginosa* (G−)], together with two species of fungus (*Candida albicans*, *Cryptococcus neoformans*). Only compound **7** was partially inhibitory towards *Staphylococcus aureus* (MRSA). All other compounds (**4**–**6** and **8**) showed no appreciable antimicrobial activity. As previously indicated, polysiphonol (**10**) was unstable and therefore could not be assessed for any biological activity. Efforts were made to re-isolate polysiphonol (**10**), but this was hindered by the lack of sufficient marine algae to permit further extraction. An attempt was also made to re-collect the alga, but *P. decipiens* is not a “common” species of South Eastern Australia, and thus far we have not been successful in obtaining a further specimen of the marine alga.

## 3. Materials and Methods

### 3.1. General Experimental

All reagents used were of an analytical reagent grade (AR reagents). All solvents used for HPLC were HPLC grade, and the water used was Milli Q Water. Specific rotations were recorded using a 1.5 mL cell on a Rudolph Research Analytical Autopol IV automatic polarimeter set to the Na 589 nm wavelength. ^1^H (500 MHz), ^13^C (125 MHz), and single irradiation NOE and TOCSY spectra were acquired in CD_3_OD and CDCl_3_ using a 500 MHz Agilent DD2 spectrometer. Two dimensional experiments that were performed included gradient correlation spectroscopy (gCOSY), heteronuclear single-quantum correlation spectroscopy with adiabatic pulses (HSQCAD), and gradient heteronuclear multiple-bond spectroscopy with adiabatic pulses (gHMBCAD). Bench top C_18_ Vaccuum Liquid Chromatography (VLC) was performed using silica gel 60 RP-18 (40–63 μm) with a 20% stepwise solvent elution beginning with 100% H_2_O to 100% MeOH and finally to 100% CH_2_Cl_2_. LC high-resolution electrospray ionisation mass spectroscopy (LC-HRESIMS) was carried out on an Agilent 6540 QTOF (ESI operation conditions of 10 L/min N_2_, 300 °C drying gas temperature, and 4000 V capillary range) coupled with an Agilent 1260 series LC (0–2 min 15% CH_3_CN/H_2_O; 18–20 min 65% CH_3_CN/H_2_O; 21–22 min 15% CH_3_CN/H_2_O run at a flow rate of 1.0 mL/min). This system utilised UV detection from an Agilent 1260 Infinity DAD. The instrument was calibrated using the “Agilent Tuning Mix” with purine as the reference compound and the Hewlett-Packard standard HP0921. LC-HRESIMS was performed using an Agilent ZORBAX Eclipse Plus (5 μ), C_18_, 150 × 4.6 mm column. ESI mass spectra were obtained on all pure compounds using a Micromass Platform II mass spectrometer equipped with an LC-10AD Shimadzu solvent delivery module (50% CH_3_CN/H_2_O at a flow rate of 0.2 mL/min). Analysis was performed in both positive and negative ionisation modes using a cone voltage between 20 and 30 V. All analytical reversed phase HPLC analyses and method developments were performed on a Dionex P680 solvent delivery system that was equipped with a PDA100 UV detector (operating software was Dionex Chromeleon, version 6.80, Sunnyvale, CA, USA). All analytical HPLC was performed on an Agilent ZORBAX Eclipse Plus (5 μ), C_18_, 250 × 4.6 mm column using a gradient method (0–2 min 10% CH_3_CN/H_2_O; 14–24 min 75% CH_3_CN/H_2_O; 26–30 min 100% CH_3_CN; and 32–40 min 10% CH_3_CN/H_2_O run at 1.0 mL/min). All semi-preparative reversed phase chromatography was performed using a Varian Prostar 210 solvent delivery system equipped with a Prostar 335 PDA detector (operated using Varian Star Workstation software, version 6.30, Sunnyvale, CA, USA) using a gradient method (0–2 min 15% CH_3_CN/H_2_O; 18–40 min 65% CH_3_CN/H_2_O run at 4.0 mL/min). All semi-preparative HPLC was performed on an Agilent ZORBAX Eclipse XDB-C_18_, 250 × 9.4 mm, 5 μm column.

### 3.2. Biological Activity

All biological activity testing was performed by the The Community for Antimicrobial Drug Discovery (CO-ADD) [26]. See supporting information document for a description of biological activity procedures and the results (Supplementary Materials S1 and S5).

### 3.3. Collection Details

The red alga *P. decipiens* was collected via SCUBA just off shore at Queenscliffe, Port Phillip Bay, Victoria, Australia at a depth of 1.5–3 m on 23 March 2016. The alga was identified by Dr. Gerald Kraft, School of Biosciences, University of Melbourne, Australia and the Tasmanian Herbarium, Sandy Bay, Tasmania, Australia. A voucher specimen (designated the code number 2016_11) is deposited at the School of Science, RMIT University.

### 3.4. Extraction and Isolation

The alga (29.9 g, wet weight) was extracted in a solvent system of 3:1 MeOH:CH_2_Cl_2_ (1 L). The crude extract was filtered and concentrated using reduced pressure. The crude extract was sequentially solvent partitioned (triturated) into CH_2_Cl_2_ (125.5 mg) and MeOH (1 g) soluble fractions. The MeOH extract was subject to C_18_ Vacuum liquid chromatography (20% stepwise elution from H_2_O to MeOH and then to CH_2_Cl_2_ and finally flushed using 0.1% trifluoroacetic acid (TFA) in MeOH). The fraction eluted with 20:80 H_2_O:MeOH was subject to reversed phase semi-preparative HPLC using a gradient method (0–2 min 15% CH_3_CN/H_2_O; 18–40 min 65% CH_3_CN/H_2_O run at 4.0 mL/min). This yielded compounds **4** (1.3 mg, 0.11%), **5** (0.4 mg, 0.03%) **6** (0.7 mg, 0.06%), **7** (1.7 mg, 0.14%), **8** (1.5 mg, 0.13%), and **10** (0.5 mg, 0.04%). Compound **8** was subsequently acetylated using a mixture of 1:1 acetic anhydride and pyridine, which was combined in a sealed vessel and left to stir for 24 h, resulting in compound (**9**).

### 3.5. Compound Data

*α-O-Methyllanosol* (**4**); isolated as a brown oil; all NMR and MS data were identical to the previously published data [18].

*Lanosol* (**5**); isolated as a brown oil; all NMR and MS data were identical to the previously published data [17].

*5-(2-Bromo-3*,*4-dihydroxy-6-(hydroxymethyl)benzyl)-3*,*4-dibromobenzene-1*, *2-diol* (**6**); isolated as a brown oil; all NMR and MS data were identical to the previously published data [18].

*5-(2-Bromo-3*, *4-dihydroxy-6-(methoxymethyl)benzyl)-3*, *4-dibromobenzene-1*, *2-diol* (**7**); isolated as a brown oil; all NMR and MS data were identical to the previously published data [16].

*Rhodomelol* (**8**); isolated as a brown oil; ${\left[\alpha \right]}_{D}^{25}$: +29.1 c 0.5, 99% MeOH; all NMR and MS data were identical to the previously published data [3,19,20].

*Acetylated rhodomelol* (**9**); to a solution of rhodomelol (**8**) in pyridine (0.6 mL) was added a 0.6 mL quantity of acetic anhydride. The reaction was left to stir at room temperature for 24 h. The resulting mixture was dried, resulting in a brown oil; ^1^H NMR (500 MHz, CD_3_OD) *δ* 7.46 (1H, *s*, H-6), 5.54 (1H, *s*, H-12), 5.40 (1H, *t*, H-11), 4.42 (1H, *t*, 9 Hz, H-10a), 4.22 (1H, *dd*, 5.5, 9, Hz, H-10b), 3.53 (1H, *d*,15.5 Hz, H-7a), 3.61 (1H, *d*, 15.5 Hz, H-7b), 2.04 (1H, *s*, H-9Ac), 2.13 (1H, *s*, H-11Ac), 2.15 (1H, *s*, H-8Ac), 2.29 (1H, *s*, H-5Ac), 2.35 (1H, *s*, H-4Ac). ^13^C NMR (125 MHz, CD_3_OD) *δ* 170.5 (C, C-11 **C**OCH_3_), 169.8 (C, C-8 **C**OCH_3_), 169.0 (C, C-9 **C**OCH_3_), 168.9 (C, C-13), 168.0 (C, C-5 **C**OCH_3_), 167.1 (C, C-4 **C**OCH_3_), 141.9 (C, C-5), 140.6 (C, C-4), 134.2 (C, C-1), 125.5 (CH, C-6), 120.5 (C, C-2), 109.3 (C, C-9), 87.8 (CH, C-12), 79.4 (C, C-8), 77.7 (CH, C-11), 75.7 (CH_2_, C-10), 39.1 (CH_2_, C-7), 19.7 (CH_3_, C-9 CO**C**H_3_), 19.1 (CH_3_, C-11 CO**C**H_3_), 19.0 (CH_3_, C-5 CO**C**H_3_), 18.8 (CH_3_, C-4 CO**C**H_3_), 18.7 (CH_3_, C-8 CO**C**H_3_).

*Polysiphonol* (**10**); isolated as an unstable brown oil; UV (extracted from PDA) λ_max_: 235 and 291 nm; ^1^H and ^13^C NMR see Table 1; LC-HRESIMS (negative ion mode) *m*/*z* 652.82879 observed for C_20_H_16_^79^Br_3_O_10_ [M − H]^+^; calcd. for C_20_H_16_^79^Br_3_O_10_ *m*/*z* 652.82991.

## 4. Conclusions

In the present study, a total of six bromophenolic compounds were isolated and characterised from the Southern Australian marine alga *P. decipiens*. This marks the first phytochemical study of this particular species of *Polysiphonia*, yielding the unprecedented natural product polysiphonol (**10**). Also reported here is the first stereochemical assignment of the previously reported natural product rhodomelol (**8**). Four other previously reported bromophenolics (**4**–**7**) were also obtained from the methanol extract of this alga. Compounds **4**–**8** were all evaluated for anti-microbial activity, however, none of the compounds showed any appreciable activity (see supporting information file).

## Figures and Tables

**Table 1:** NMR data (500 MHz, CD_3_OD) for polysiphonol (**10**).

Position	Carbon, Type ^a^	Proton, Mult. (*J* in Hz)	gHMBCAD
1	125.9, C ^b^		
2	129.4, C ^b^		
3	114.7, C ^c^		
4	142.4, C ^d^		
5	143.8, C ^d^		
6	117.5, CH	6.92, s	2, 4, 5, 7
7a	36.9, CH_2_	2.93, d (15.0)	1, 2, 6, 8, 9, 13 ^W^
7b		2.78, d (15.0)	1, 2, 6, 8, 9
8	79.1, C		
9	107.4, C		
10a	74.9, CH_2_	4.19, dd (5.5, 9.5)	9, 12, 11 ^W^
10b		4.03, dd (3.0, 9.5)	11 ^W^
11	74.2, CH	4.36, bs	10
12	86.8, CH	4.36, bs	10, 13
13	175.6, C		
1a’	39.3, CH_2_	4.24, d (17.5)	1 ^W^, 2, 2′ ^W^, 7′ ^W^
1b’		4.08, d (17.5)	1, 2, 2′, 3′, 7′
2′	131.3, C		
3′	114.7, C ^c^		
4′	114.7, C ^c^		
5′	142.5, C ^e^		
6′	144.9, C ^e^		
7′	113.8, CH	5.97, s	3′ ^W^, 5′, 6′
4-OH		ND	
5-OH		ND	
8-OH		ND	
9-OH		ND	
11-OH		ND	
5′-OH		ND	
6′-OH		ND	

^a^ carbon assignments are based on HSQC with adiabatic pulses (HSQCAD) and gradient HMBCAD (gHMBCAD) NMR experiments. ^W^ indicates weak or long range correlation. ND indicates signal not detected. ^b–e^ indicates signals may be interchangeable.

## Supplementary Materials

The following are available online at https://www.mdpi.com/1660-3397/17/9/497/s1, S1 CO-ADD antimicrobial testing procedures, S2 CO-ADD antifungal testing procedures, S3 CO-ADD antibiotic standard preparations, S4 CO-ADD results, S5 Supporting NMR spectra.
