# Supplementary material for: Bromophenolics from the Red Alga Polysiphonia decipiens
Source: Mar Drugs. 2019 Aug 26;17(9):497. doi: 10.3390/md17090497 (PMC6780756; doi:10.3390/md17090497)
Supplement: Supplementary file 1 [file marinedrugs-17-00497-s001.pdf]

Bromophenolics from the Red Alga *Polysiphonia decipiens*

James Lever[1], Grace Curtis[1,2], Robert Brkljača[1], Sylvia Urban[1,*]

1 School of Science (Applied Chemistry and Environmental Science), RMIT University, GPO Box 2476V Melbourne, Victoria 3001, Australia.

2 Now at Medicinal Chemistry, Monash Institute of Pharmaceutical Sciences, Monash University (Parkville Campus), 381 Royal Parade, Parkville, Victoria 3052, Australia.

* Correspondence: sylvia.urban@rmit.edu.au

Contents:

S1. CO-ADD antimicrobial testing procedures.

S2. CO-ADD antifungal testing procedures.

S3. CO-ADD antibiotic standard preparations.

S4. CO-ADD results.

S5. Supporting NMR Spectra.

Supporting Information:

S1. CO-ADD antimicrobial testing procedures.

All bacteria were cultured in Cation-adjusted Mueller Hinton broth (CAMHB) at 37 °C overnight. A sample of each culture was then diluted 40-fold in fresh broth and incubated at 37 °C for 1.5-3 h. The resultant mid-log phase cultures were diluted (CFU/mL measured by OD_{600}), then added to each well of the compound containing plates, giving a cell density of $5x10^5$ CFU/mL and a total volume of 50 μL. All the plates were covered and incubated at 37 °C for 18 h without shaking.

Inhibition of bacterial growth was determined measuring absorbance at 600 nm (OD_{600}), using a Tecan M1000 Pro monochromator plate reader. The percentage of growth inhibition was calculated for each well, using the negative control (media only) and positive control (bacteria without inhibitors) on the same plate as references. The significance of the inhibition values was determined by modified Z-scores, calculated using the median and MAD of the samples (no controls) on the same plate. Samples with inhibition value above 80% and Z-Score above 2.5 for either replicate (n=2 on different plates) were classed as actives. Samples with inhibition values between 50 - 80% and Z-Score above 2.5 for either replicate (n=2 on different plates) were classed as partial actives. Samples with inhibition values between 50 - 80% and Z-Score above 2.5 for either replicate (n=2 on different plates) were classed as partial actives.

Table 1: Microbial Strains Used for assays.

ID	Batch	Organism	Strain	Description
GN_001	02	*Escherichia coli*	ATCC 25922	FDA control strain
GN_003	02	*Klebsiella pneumoniae*	ATCC 700603	MDR
GN_034	02	*Acinetobacter baumannii*	ATCC 19606	Type strain
GN_042	02	*Pseudomonas aeruginosa*	ATCC 27853	Quality control strain
GP_020	02	*Staphylococcus aureus*	ATCC 43300	MRSA
FG_001	01	*Candida albicans*	ATCC 90028	CLSI reference
FG_002	01	*Cryptococcus neoformans*	ATCC 208821	H99 – Type strain

S2. CO-ADD antifungal testing procedures.

Fungi strains were cultured for 3 days on Yeast Extract-Peptone Dextrose (YPD) agar at 30 °C. A yeast suspension of 1 x 10^6 to 5 x 10^6 CFU/mL (as determined by OD_{530}) was prepared from five colonies. The suspension was subsequently diluted and added to each well of the compound-containing plates giving a final cell density of fungi suspension of 2.5 x10^3 CFU/mL and a total volume of 50 µL. All plates were covered and incubated at 35 °C for 24 h without shaking.

Growth inhibition of *C. albicans* was determined measuring absorbance at 530 nm (OD_{530}), while the growth inhibition of *C. neoformans* was determined measuring the difference in absorbance between 600 and 570 nm ($OD_{600\text{-}570}$), after the addition of resazurin (0.001% final concentration) and incubation at 35 °C for additional 2 h. The absorbance was measured using a Biotek Synergy HTX plate reader. The percentage of growth inhibition was calculated for each well, using the negative control (media only) and positive control (fungi without inhibitors) on the same plate. The significance of the inhibition values was determined by modified Z-scores, calculated using the median and MAD of the samples (no controls) on the same plate. Samples with inhibition value above 80% and Z-Score above 2.5 for either replicate (n=2 on different plates) were classed as actives. Samples with inhibition values between 50 - 80% and Z-Score above 2.5 for either replicate (n=2 on different plates) were classed as partial actives.

S3. CO-ADD antibiotic standard preparations.

Colistin and Vancomycin were used as positive bacterial inhibitor standards for Gram- negative and Gram-positive bacteria, respectively. Fluconazole was used as a positive fungal inhibitor standard for C. albicans and C. neoformans. The antibiotics were provided in 4 concentrations, with 2 above and 2 below its MIC value, and plated into the first 8 wells of column 23 of the 384-well NBS plates.

The quality control (QC) of the assays was determined by the antimicrobial controls and the Z′-factor (using positive and negative controls). Each plate was deemed to fulfil the quality criteria (pass QC), if the Z′-factor was above 0.4, and the antimicrobial standards showed full range of activity, with full growth inhibition at their highest concentration, and no growth inhibition at their lowest concentration.

S4. CO-ADD results.

Table 2

CO-ADD antimicrobial assay results.

Comp ID	Structure	% Inhibition of Microbial Species*							Conc.
		Sa	Ec	Kp	Pa	Ab	Ca	Cn	
C0369588	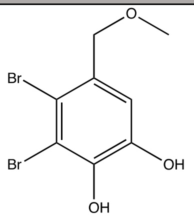	38.05	16.17	3.21	3.47	20.6	9.65	-0.07	32 µg/mL
C0369589	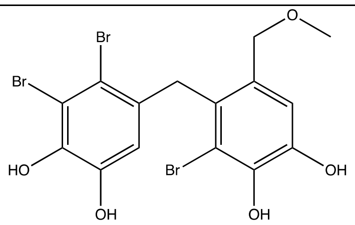	71.81	19.47	19.88	4.89	46.12	7.64	-14.08	32 µg/mL
C0369585	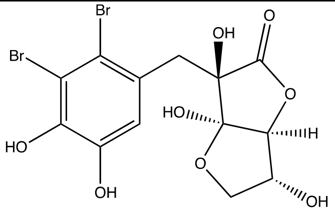	5.75	10.76	12	10.1	-64.43	0.44	18.47	32 µg/mL
C0369586	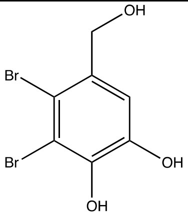	47.78	21.74	-8.15	12.02	-86.31	41.98	17.68	32 µg/mL
C0369587	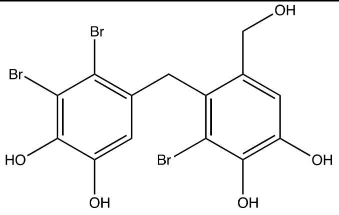	57.03	16.41	6.06	3.1	-2.73	5.53	-3.09	32 µg/mL

* Sa: MRSA, Ec: *E. coli*, Kp: *Klebsiella pneumoniae*, Pa: *Pseudomonas aeruginosa*, Ab: *Acinetobacter baumannii*, Ca: *Candida albicans*, Cn: *Cryptococcus neoformans.*

Inhibition

Percentage growth inhibition of an individual sample is calculated based on Negative controls (media only) and Positive controls (bacteria/fungal media without inhibitors). Please note negative inhibition values indicate that the growth rate (or OD600) is higher compared to the Negative Control (Bacteria/fungi only, set to 0% inhibition). The growth rates for all bacteria and fungi has a variation of -/+ 10 %, which is within the reported normal distribution of bacterial/fungal growth. Any significant variation (or outliers/hits) is identified by the modified Z-score, and actives are selected by a combination of inhibition value and Z-Score.

S5. Supporting NMR Spectra.

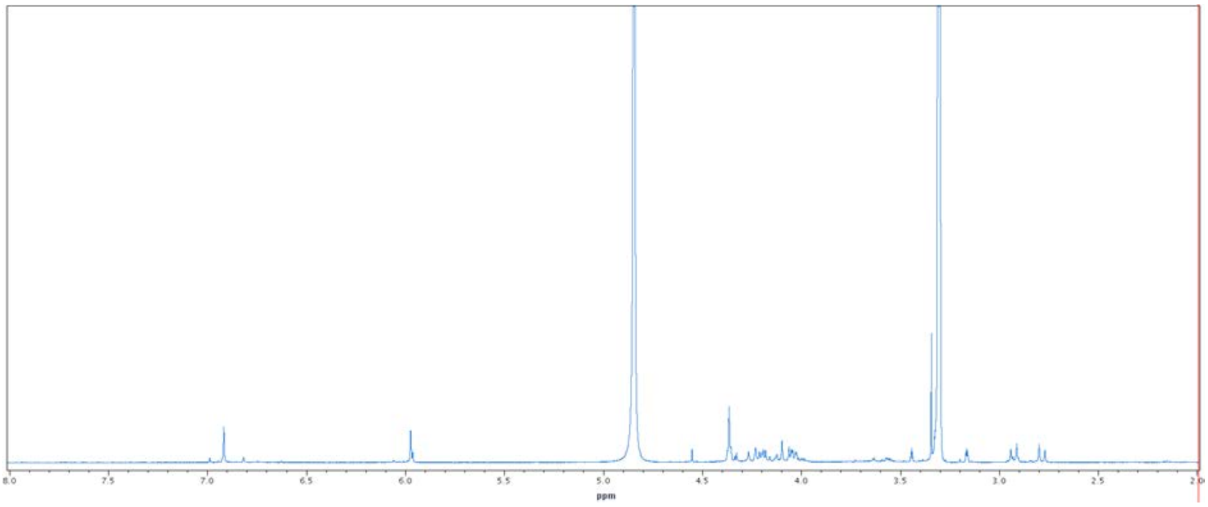

Figure 1. ^{1}H NMR spectrum of Polysiphonol **(10)**.

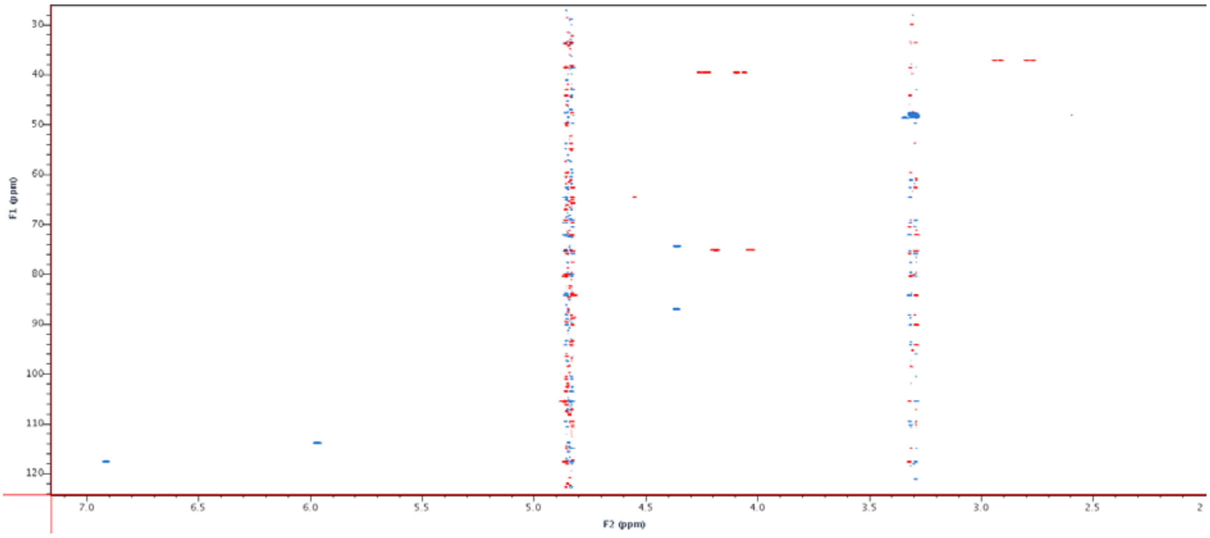

Figure 2. HSQCAD spectrum of Polysiphonol **(10)**.

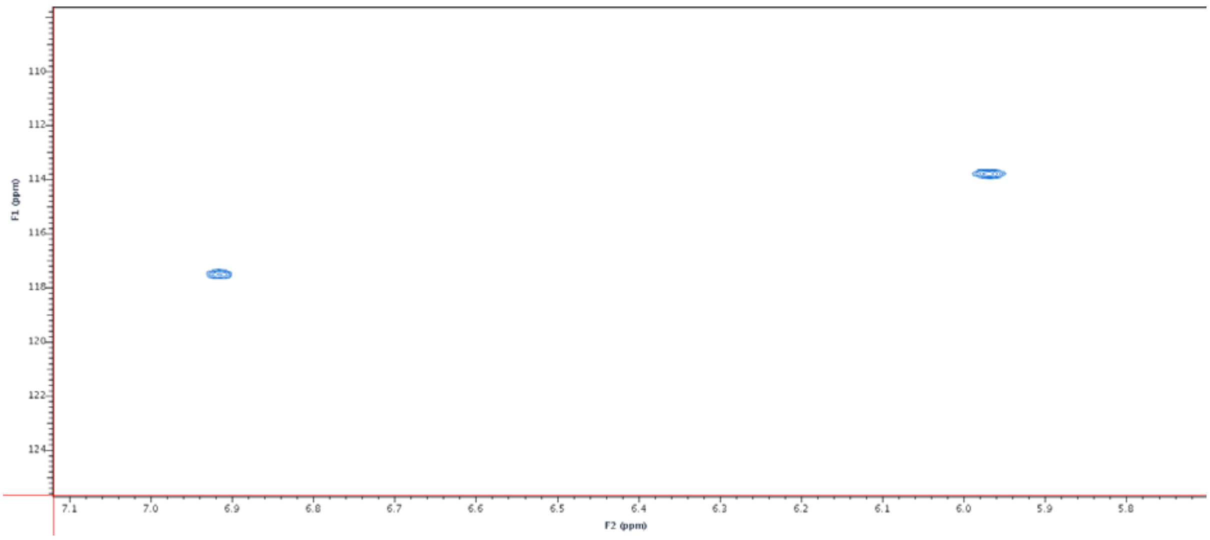

Figure 3. HSQCAD (downfield zoom) spectrum of Polysiphonol **(10)**.

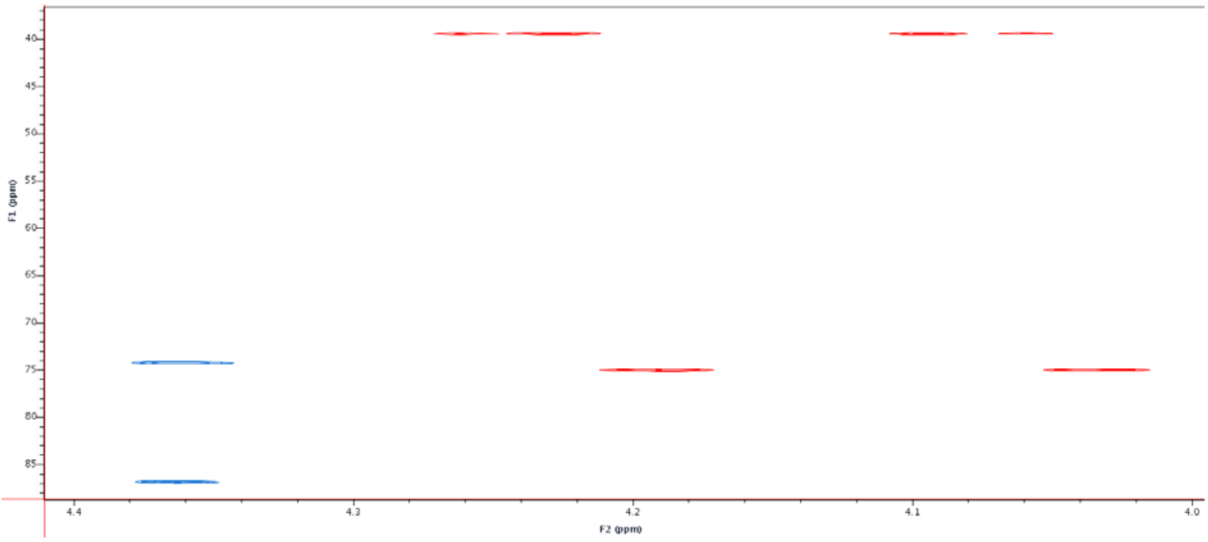

Figure 4. HSQCAD (midfield zoom) spectrum of Polysiphonol **(10)**.

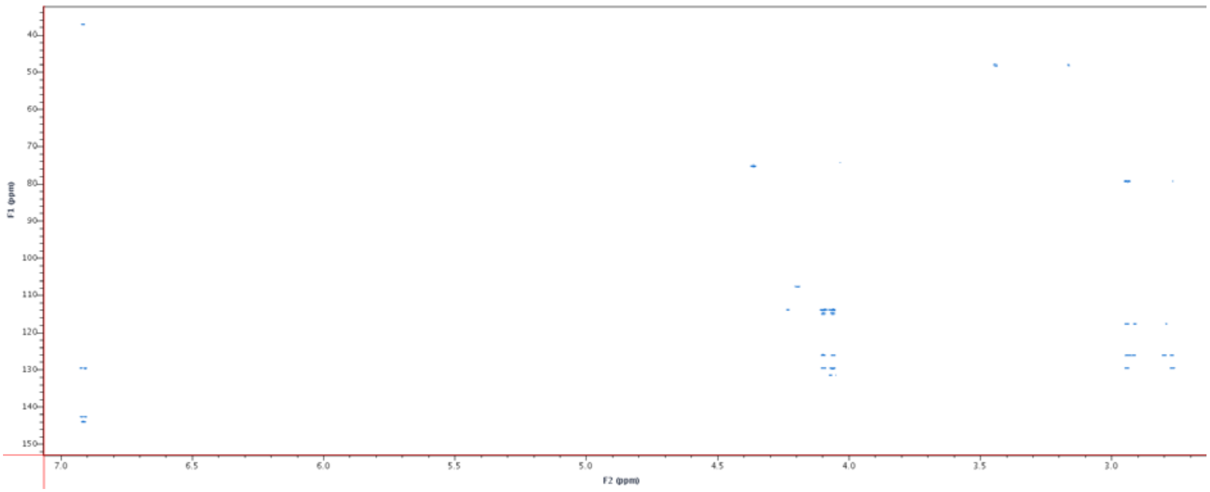

Figure 5. gHMBCAD spectrum of Polysiphonol **(10)**.

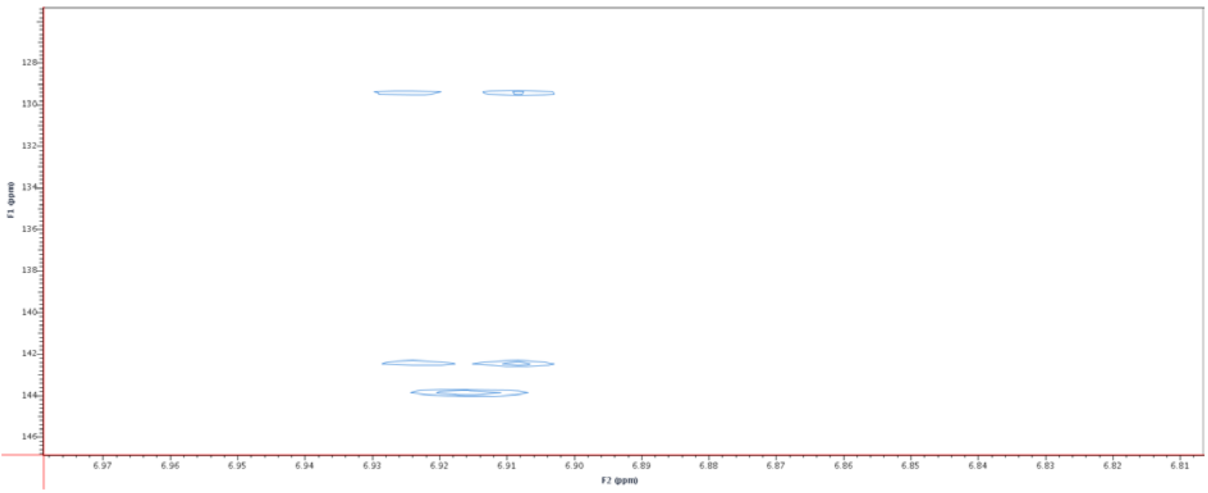

Figure 6. gHMBCAD (downfield zoom) spectrum of Polysiphonol **(10)**.

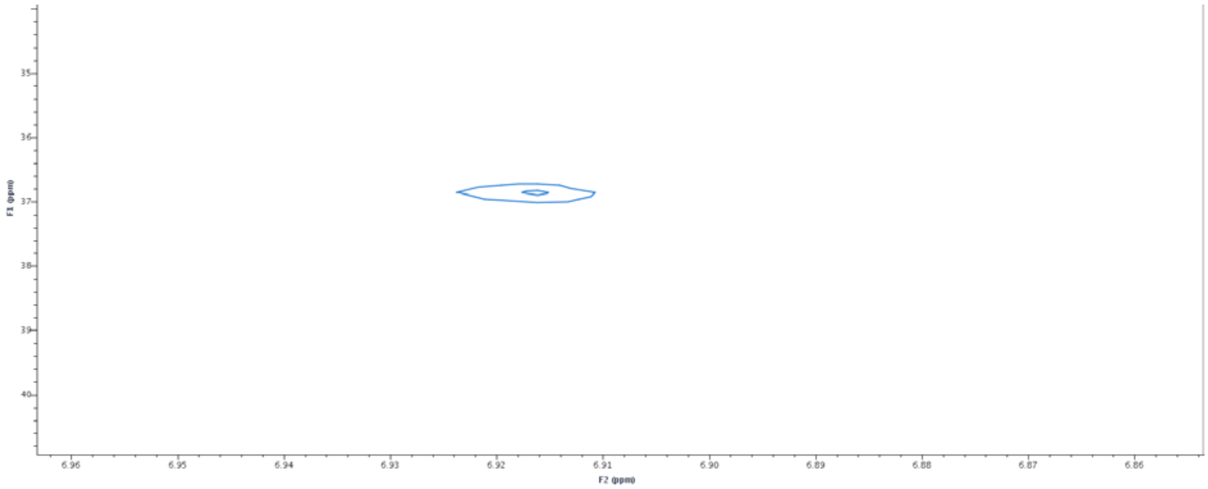

Figure 7. gHMBCAD (downfield zoom1) spectrum of Polysiphonol **(10)**.

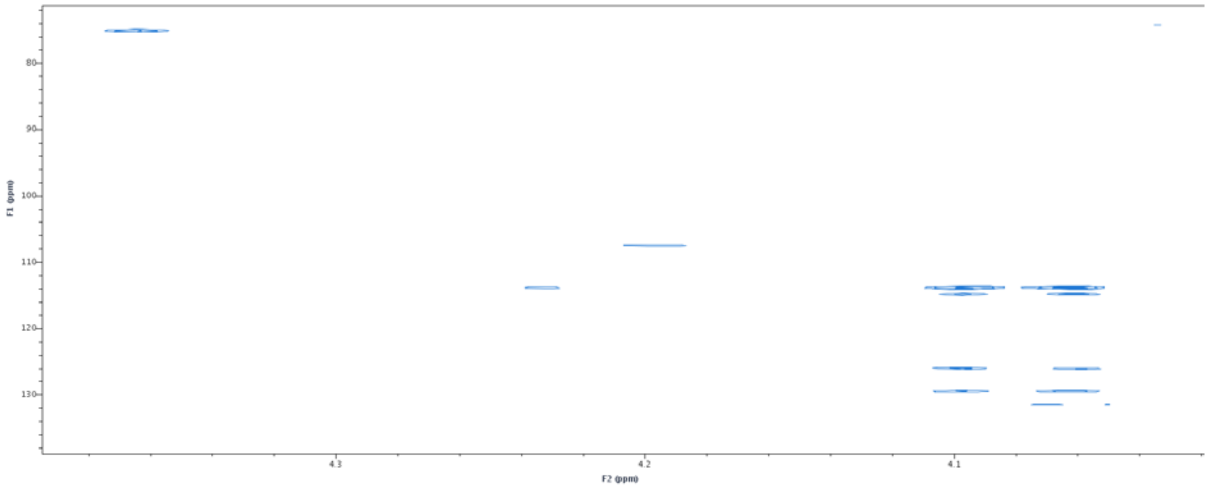

Figure 8. gHMBCAD (midfield zoom) spectrum of Polysiphonol **(10)**.

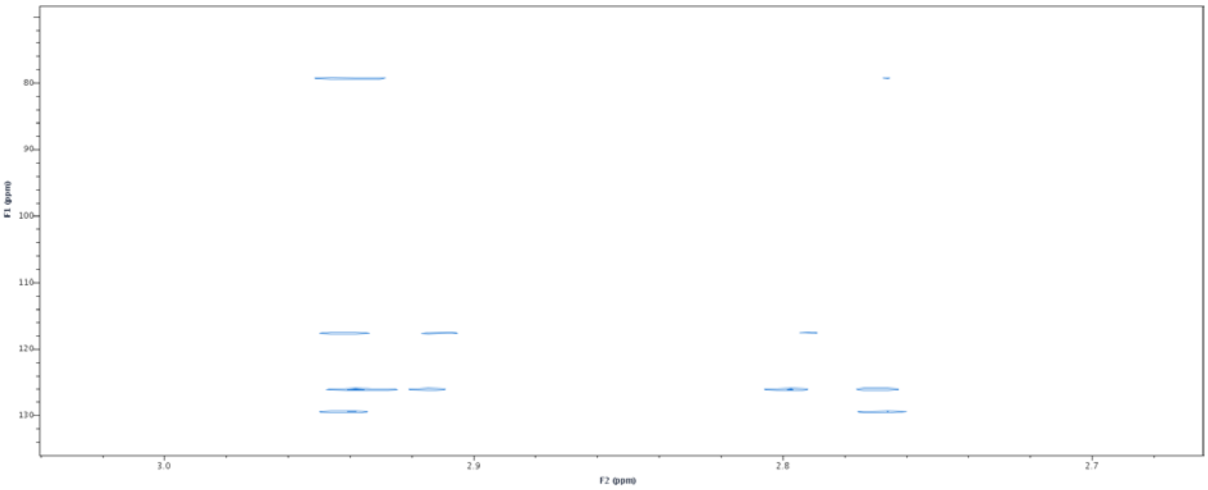

Figure 9. gHMBCAD (midfield zoom1) spectrum of Polysiphonol **(10)**.

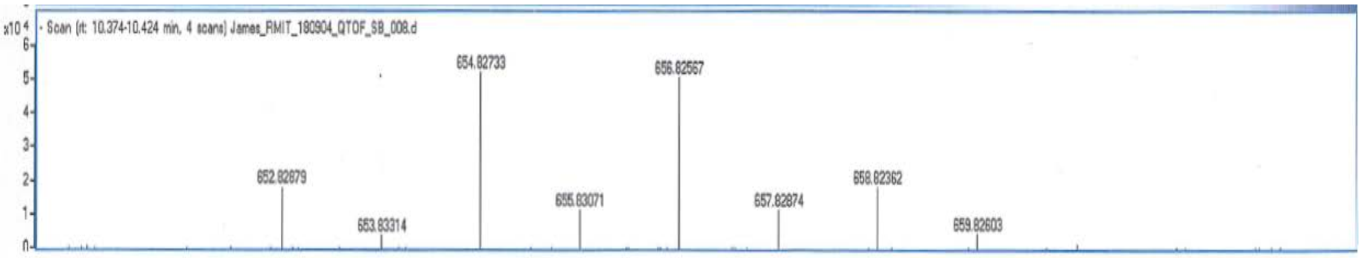

Figure 10. LC-HRESIMS (expansion) spectrum of Polysiphonol **(10)**
